# Integrated morphological, physiological, and transcriptomic analyses uncover the mechanisms of waterlogging tolerance in *Sorghum bicolor* (L.)

**DOI:** 10.3389/fpls.2025.1706603

**Published:** 2025-12-08

**Authors:** Ruidong Zhang, Haiyun Chen, Xiang Li, Minli Wang, Yu Luo, Wenda Jiao, Jiarong Chang, Xiong Cao, Jun Feng

**Affiliations:** 1 Institute of Industrial Crops, Shanxi Agricultural University, Taiyuan, China; 2 College of Agronomy, Shanxi Agricultural University, Taigu, Jinzhong, China; 3 College of Biological Science and Technology, Taiyuan Normal University, Jinzhong, China

**Keywords:** sorghum, RNA-Seq, adventitious roots, anaerobic respiratory enzymes, WGCNA

## Abstract

Sorghum (*Sorghum bicolor* L.) is a vital global crop often cultivated in waterlogging-prone regions. However, its productivity is severely limited by waterlogging stress, which inhibits growth and significantly reduces yields. To address this, we investigated the physiological and molecular mechanisms of waterlogging tolerance by comparing a tolerant variety ‘S208’ with a sensitive one ‘S015’. After 12 days of waterlogging, ‘S208’ exhibited markedly less reduction in plant height, stem thickness, and leaf area. It also developed more and longer adventitious roots and, after 6 days, formed a significantly higher proportion of aerenchyma Physiological assays revealed that the activities of key anaerobic respiratory enzymes, including pyruvate decarboxylase (PDC), lactate dehydrogenase (LDH), and alcohol dehydrogenase (ADH), were consistently higher in ‘S208’. Transcriptomic analysis 24 hours post-waterlogging identified differentially expressed genes (DEGs) enriched in energy metabolism, hormone regulation, and cell wall modification. Weighted gene co-expression network analysis (WGCNA) further highlighted the predominant regulatory roles of AP2/ERF, BHLH and WRKY transcription factor families. Our finding demonstrate that the superior waterlogging tolerance of ‘S208’ is atrributed to integrated morphological, phsiological, and transcriptional adaptations, providing valuable insights for breeding resilient sorghum varieties.

## 1 Introduction

Waterlogging, a prevalent and detrimental abiotic stress, poses a significant threat to global crop production. Climate change intensification has increased the frequency of extreme weather, leading to more frequent waterlogging in agricultural areas (Ngumbi, 2025). It is estimated that approximately 12% of the world’s arable land is affected by excessive water accumulation, resulting in an estimated 20% reduction in global crop yields (Chadalavada et al., 2021). This scenario not only threatens food security but also underscores the urgent need for agricultural research focused on mitigation strategies.

Sorghum (*Sorghum bicolor* L.), the fifth most important cereal crop globally, is valued for its versatility (Dahlberg, 2019). It serves as a crucial source of food, forage, brewing materials, and industrial raw materials (Vanamala et al., 2018). Althogh sorghum exhibits adaptability to marginal environments such as drought-prone soils, its cultivation in waterlogged regions-including parts of China is severely limited by its sensitivity to waterlogging (Zhang R. et al., 2023). This stress disrupts multiple physiological processes in sorghum. For instance, it reduces the oxygen availability in root aerenchyma (Xu et al., 2023), impairs photosynthesis (Zhang et al., 2019), and ultimately constrains yield potential (Zhang R. et al., 2023). As the primary organs affected, roots experience hypoxia, which disrupts respiration and energy production (Sarma et al., 2023). Consequently, energy deficits inhibit root elongation and function, compromising water and nutrient uptake (Shao et al., 2024). Furthermore, anaerobic respiration under waterlogging generates toxic substances (e.g. ethanol and lactic acid) that interfere with essential metabolic pathways, including glycolysis, the tricarboxylic acid (TCA) cycle, and oxidative phosphorylation, further suppressing plant growth and photosynthesis (Xu et al., 2023).

To cope with waterlogging stress, plants have evolved a range of morphological and physiological adaptations. Recent studies have extensively documented these mechanisms in waterlogging-tolerant species (Xu et al., 2023; Li et al., 2024). A key adaptative response is the formation of adventitious roots (ARs) (Li et al., 2022). Under waterlogging conditions, primary roots often lose their capacity for efficient water and nutrient uptake, and ARs develop to compensate (Manik et al., 2022). For example, waterlogging-tolerant barley genotypes show a pronounced increase in AR formation under stress (Manik et al., 2022). Another crucial adaptation is aerenchyma formation. Rice, which exhibits greater waterlogging tolerance than dryland crops like barley and wheat, benefits from constitutive aerenchyma in its root system (Pan et al., 2021). Under hypoxia, the proportion of aerenchyma increases significantly, as also observed in barley under waterlogging stress (Luan et al., 2023). Metabolic adjustments are equally important, when aerobic respiration is inhibited, anaerobic pathways-such as ethanol fermentation, lactate fermentation, and glycolysis are activated to sustain energy supply. Key enzymes involved in these pathway, including pyruvate decarboxylase (PDC) and ethanol dehydrogenase (ADH), show activity levels closely correlated with waterlogging tolerance (Zhao et al., 2024). Enzymes related to carbohydrate metabolism, such as sucrose synthase and trehalose-6-phosphate phosphatase, also contribute to plant responses under waterlogging (Zeng et al., 2024).

Transcriptional regulation plays a central role in plant adapation to waterlogging. Transcription factors (TFs) from families such as MYB, ERF, WRKY, NAC, and bZIP regulate genes involved in hypoxia signaling, reactive oxygen species (ROS) scavenging, and root morphological adaptations across diverse crops (Zhang et al., 2024). For example, ERF-family genes like *SUBMERGENCE1A* in rice (Alpuerto et al., 2016) and *ZmEREB180* in maize (Yu et al.) modulate anaerobic metabolism, gibberellin degradation, and adventitious root development. WRKY TFs (e.g. *WRKY22* and *WRKY33*) enhance waterlogging tolerance, often by interacting with other proteins to alleviate ROS accumulation (Hsu et al., 2013; Tang et al., 2021). Additionally, metabolic genes such as *alcohol dehydrogenase* (*ADH*) in barley (Luan et al., 2023), phenylalanine metabolism and biosynthesis genes in *Cynodon dactylon (*Zhao et al., 2024), and starch/sucrose metabolism genes in peanut (Zeng et al., 2024) have been implicated in waterlogging responses.

Despite these advances, the root-specific physiological and transcriptional mechanisms underlying waterlogging tolerance in sorghum remain poorly understood. This knowledge gap hinders the development of tolerant sorghum varieties through targeted breeding. To address this, our study comprehensively evaluates root morphology, physiological traits, and transcriptome profiles of two sorghum varieties with contrasting waterlogging tolerances under waterlogged conditions. By integrating morphological, physiological, and RNA-seq analyses, we aim to elucidate the molecular and physiological basis of sorghum’s adaptation to waterlogging stress. Our findings will not only enhance the understanding of sorghum genetics but also provide actionable insights for breeding programs designed to improve waterlogging tolerance, thereby supporting global food security in water-vulnerable agricultural regions.

## 2 Materials and methods

### 2.1 Plant materials and waterlogging treatment

The experiments were conducted from June to October 2024. In the pre-experiment, we evaluated the waterlogging tolerance of 100 sorghum varieties at the five-leaf stage. We imposed waterlogging stress on these varieties and measured phenotypic data such as plant height, number of adventitious roots, and biomass, comparing the phenotypic data of these varieties with that of the control group. Based on these evaluations, two sorghum varieties, ‘S208’ (waterlogging tolerance) and ‘S015’ (waterlogging sensitivity), were selected for this study. Seeds of ‘S208’ and ‘S015’ were sterilised with 0.1% sodium hypochlorite. The seeds were sown in pots with a diameter of 17 cm and a volume of 4L. The potting substrates, a well-mixed blend of peat soil and vermiculite in a ratio of 3:1, were used for planting. The temperature in the green-house was maintained at 25-30°C, and the light intensity was set at 400-600 μmol/m²/s. The soil moisture content of the control group was kept at 65-75%. Fourteen days after seedling emergence, the plants were subjected to waterlogging treatment. To ensure the relative consistency of the test material, we assessed the growth of sorghum plants using multiple parameters. These parameters included plant height (measured from the base of the plant to the tip of the tallest leaf), leaf color (healthy, green without significant yellowing or browning) and expansion (well-expanded leaves with normal shape and size). Only one robustly growing plant, determined by these growth parameters, was retained from each pot, while excess sorghum seedlings were removed during the treatment. Sorghum plants without waterlogging treatment were used as control (CK). In short, the pots containing the plants were placed in plastic containers. These plastic containers had a length of 90 cm, a width of 60 cm, and a height of 25 cm. They were filled with water, and the water level is set at 3 cm above the soil surface of the pots. Sorghum root samples were collected 24 hours after waterlogging for transcriptome analysis. At 0, 3, 6, 9 and 12th day waterlogging, the root samples were collect for physiological indicators measured.

### 2.2 Phenotype observation after waterlogging

Morphological data were collected from randomly selected sorghum plants at 0d, 3d, 6d, 9d and 12d of waterlogging treatment. And each sample was subjected to de-termination of plant height, leaf length, leaf width, Soil and plant analyzer develop-ment (SPAD) value, and adventitious root number and length. At 6 d after waterlogging, the roots of newborn adventitious roots (referred to as adventitious roots growing after substitution waterlogging) and control plants were sampled after waterlogging treatment, respectively, and the roots were cleaned, and then the apical 1 cm was removed with a sharp blade, and then 5 cm of the roots were cut off and fixed in formaldehyde-acetic acid-ethanol (FAA) solution. Paraffin sections were prepared according to Luan et al (Luan et al., 2023). The samples were then dehydrated using a series of ethanol concentrations (20%, 40%, 60%, 80%, 90%, 95% and 100%, for 15 minutes each). Tissues were then infiltrated and embedded in a special kind of resin called SPI low viscosity Spurr resin (Electron Microscopy Sciences, USA), using a chemical called propylene oxide instead of ethanol. Sections 1 μm thick were cut with a glass knife on a Leica Ultracut R (Leica Microsystems, Germany), stained with 0.5% methyl violet for 10 min, and then photographed under a light microscope (Leica, Germany). The areas of the root aerenchyma and total root cross-section were measured using Image pro plus (IPP) software (Media Cybernetics, USA).

### 2.3 Determination of anaerobic respiratory enzyme activity

Root samples (0.5 g per replicate) were immediately frozen in liquid nitrogen and stored at −80°C for enzyme activity measurements. Crude enzymes extract followed the methods described as previously (Liu et al., 2023). The sample were ground to a powder in an ice bath to form a slurry and boiled in a water bath for 10 min. Mix the sample powder and extraction solution at a ratio of 1:10, and centrifuge at 4°C 8000 g for 10 minutes. The pyruvate decarboxylase (PDC, EC 4.1.1.1), alcohol dehydrogenase (ADH, EC 1.1.1.1), and lactate dehydrogenase (LDH, EC 1.1.1.17) activities were measured using corresponding activity assay kits (Beijing Boxbio Science & Technology co. Ltd), according to the manufacturer’s protocol. using determination kits (Beijing Boxbio Science & Technology Co. Ltd), according to the manufacturer’s protocol. The results were expressed as U/g fresh weight.

### 2.4 RNA extraction, library preparation, RNA sequencing, and sequence assembly

A total of 12 samples were gathered from two distinct sorghum varieties, labeled ‘S208’ and ‘S015’, for the purpose of extracting total RNA. These samples originated from three separate biological replicates, half grown under standard control conditions and the other half subjected to a 24-hour waterlogging stress regimen. The CTAB extraction technique, as detailed by Asif et al (Asif et al., 2000), was employed to isolate total RNA from the root tissues. The total RNA from every sample was extracted using the RNAprep Pure Plant Plus Kit (Tiangen, China). First, the root tissue was collected and frozen in liquid nitrogen. The frozen tissue was ground into powder in a pre-cooled mortar with continuous addition of liquid nitrogen. The powder was transferred to a pre-warmed RNase-free tube, and CTAB extraction buffer (2% CTAB, 100 mM Tris-HCl pH 8.0, 20 mM EDTA pH 8.0, 1.4 M NaCl, 2% PVP-40, with 0.2% β-mercaptoethanol added freshly) was added at a ratio of 1 mL per 0.1 g of tissue. It was mixed well and incubated at 65°C for 10–15 min, being inverted gently every 2–3 min. Then, an equal volume of chloroform:isoamyl alcohol (24:1) was added, and the tube was inverted gently. After that, it was centrifuged at 12,000×g for 15 min at 4°C, and the upper aqueous phase was transferred to a new tube. 0.6–1 volume of isopropanol was added, and it was incubated at −20°C for at least 30 min or overnight. Next, it was centrifuged at 12,000×g for 15 min at 4°C, the supernatant was removed, the pellet was washed with 70% ethanol, and it was centrifuged again at 7,500×g for 5 min at 4°C to remove the supernatant. The pellet was air-dried for 5–10 min and dissolved in the Tiangen elution buffer (30-50 μL), and then incubated at 55-60°C for 10–15 min. After successful extraction, RNA was dissolved by adding 50 μL of DEPC-treated water. The RNA quality and integrity were assessed using a Nanodrop 2000 spectrophotometer (Ther-mo Fisher Scientific, Wilmington, DE, USA), a Qubit 2.0 fluorometer (Life Technologies, Carlsbad, CA, USA), and an Agilent 2100 bioanalyzer (Agilent Technologies, Santa Clara, CA, USA). After total RNA extraction, the rRNA was removed using the Ribo ZeroTM Magnetic Kit (Epicentre, Madison, WI, USA) to enrich for mRNA, which was then reverse-transcribed into cDNA using random primers. The cDNA fragments were purified using the QiaQuick PCR Purification Kit (Qiagen, Venlo, the Netherlands), followed by end repair, the addition of A bases, and ligation to Illumina sequencing adapters. Gel electrophoresis was performed to select the appropriate size range of the ligated products, followed by PCR amplification. Sequencing was conducted using the Illumina NovaSeq 6000 platform (Gene Denovo Biotechnology Co., Guangzhou, China). After quality checks, the libraries were pooled and sequenced on an Illumina platform to produce 150 bp paired-end reads. The sequencing process involves simultaneous synthesis and sequencing, where fluorescently labeled dNTPs are incorporated into extending complementary strands, emitting fluorescence detected by the sequencer and converted into sequence information by computer software. Library construction and RNA sequencing analysis (RNA-seq) were performed at Metware Co., Ltd., Wu-han, China.

### 2.5 RNA-Seq data analysis

The raw reads obtained from sequencing were processed to remove those containing adapters and those entirely composed of adenine (A) bases. In addition, low-quality data (where more than 50% of the base quality values Q ≤ 20) was filtered out. Through these steps, high-quality clean reads were obtained. The remaining clean reads were mapped to the Sorghum bicolor reference genome sequence (Sorghum_bicolor_NCBIv3) using Tophat2 software (Kim et al., 2013). Gene expression levels were estimated using FPKM values (fragments per kilobase of exon per million fragments mapped) by the Cufflinks software (Trapnell et al., 2012). Differentially expressed genes (DEGs) in the RNA-seq data between the waterlogging treatment and control groups were identified using a DEGseq analysis. The DEGs were screened further based on whether |log_2_ Fold Change| >= 1 and FDR < 0.05. Differentially expressed genes (DEGs) underwent functional enrichment analysis. Using BLAST, we mapped DEGs to the Gene Ontology (GO) database (http://www.geneontology.org/) for GO enrichment analysis and calculated the gene count per GO term. Meanwhile, for pathway enrichment, we used BLAST to align DEGs against the Kyoto Encyclopedia of Genes and Genomes (KEGG) web server (http://www.kegg.jp/), identifying significantly enriched KEGG path-ways (Kanehisa et al., 2016; Young et al., 2016).

### 2.6 Weighted gene co-expression network analysis

WGCNA is a methodology that has been developed in accordance with a well-established protocol (Liu et al., 2024). In summary, WGCNA is a method for analysing gene expression patterns across multiple samples. The underlying concept of WGCNA is that of scale-free network distribution, whereby the correlation coefficients of the expression matrices are weighted so that highly co-evolved genes are assigned to the same gene clusters throughout the network. This process then enables entire genes to be assigned to multiple modules.

### 2.7 Quantitative real-time PCR validation

The authenticity of the DEGs screened via RNA sequencing was verified via qRT-PCR, and eight DEGs were selected for further validation via qRT-PCR. Primers were designed using Primer Premier 5.0 software, and all the primers used in the study are listed in **Supplementary Table S2** of the Appendix. GAPDH was used as the internal reference gene (Sudhakar Reddy et al., 2016). The qPCR conditions were as follows: initial denaturation at 95°C for 30 s, followed by 40 cycles of 95°C for 5s and 60°C for 30s. The relative quantification method (2^^-ΔΔCt^) was used for expression analysis. All the qRT-PCR experiments were performed with three biological replicates and three technical replicates. The mean values of these technical replicates were then used as the representative data points for the respective biological replicates in our subsequent statistical.

### 2.8 Statistical analysis

Student’s t-tests were carried out using SPSS software (v.19.0) to determine statistically significant differences between treatments, with * and ** indicating significant differences at p < 0.05 and p < 0.01, respectively.

## 3 Results

### 3.1 Morphological and physiological analysis of different sorghum varieties under waterlogging treatment

**Figure 1:**
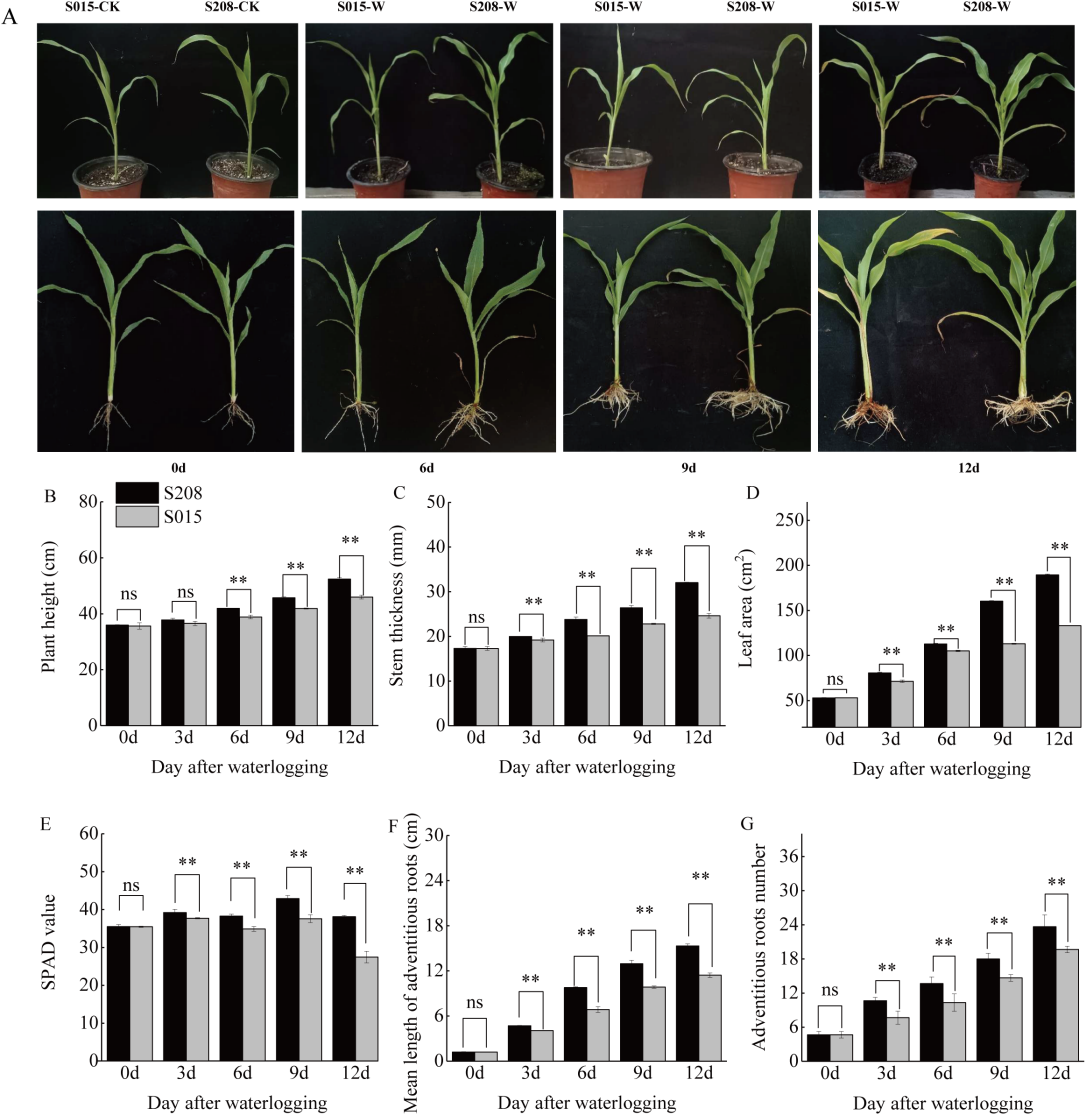
Morphological and physiological responses of two sorghum genotypes, ‘S208’ and ‘S015’, under waterlogging stress during the seedling stage. **(A)** Growth performance. **(B-G)** Statistics analysis of plant height, stem thickness, leaf area, SPAD value, mean adventitious root length, and number of adventitious roots. Data represent by mean ± standard deviation (SD) with three biological replicates. Student’s t-test was used to compare the two genotypes, with ** indicating significant differences at *p < 0.01*.

The growth and developmental responses of two sorghum genotypes, ‘S208’ and ‘S015’, were evaluated under waterlogging stress at 0, 3, 6, 9 and 12 days after treatment. Both genotypes exhibited pronounced characterized by leaf wilting and premature senescence (**Figure 1A**). However, genotypes-dependent in tolerance became apparent as early as 3 days after treatment (except for plant height), and the superiority of ‘S208’ became increasingly evident with prolonged stress exposure. After 12 days of waterlogging, ‘S208’ demonstrated stronger tolerance compared to ‘S015’, as evidenced by better performance in multiple morphological and physiological parameters.

Specifically, after 3 days of waterlogging, ‘S208’ already exhibited significant advantages over ‘S015’ in all measured parameters except plant height. At this stage, the stem thickness, leaf area, and SPAD value of ‘S208’ were 4.17%, 13.00%, and 3.98% higher than those of ‘S015’, respectively (*p < 0.05*). As waterlogging duration extended, the phenotypic and physiological differences between the two genotypes became more pronounced. By 12 days, ‘S208’ surpassed ‘S015’ by 14.01% in plant height, 31.31% in stem thickness, 42.21% in leaf area, and 41.20% in SPAD value. Adventitious root formation is a key adaptative response to waterlogging stress. Throughout the treatment period, ‘S208’ consistently developed more adventitious roots with greater lengths compared to ‘S015’. From 3 to 12 days of waterlogging, the number of adventitious roots increased by 128.57% to 407.14%, and by 64.28% to 321.42% in ‘S015’, relative to their respective control groups. Moreover, the average adventitious roots length of ‘S015’ was consistently shorter than that of ‘S208’ by 14.38%, 25.31%, 25.42% and 27.92% on days 3, 6, 9 and 12, respectively (**Figures 1B–G**).

### 3.2 Anatomical analysis of different sorghum varieties under waterlogging treatment

**Figure 2:**
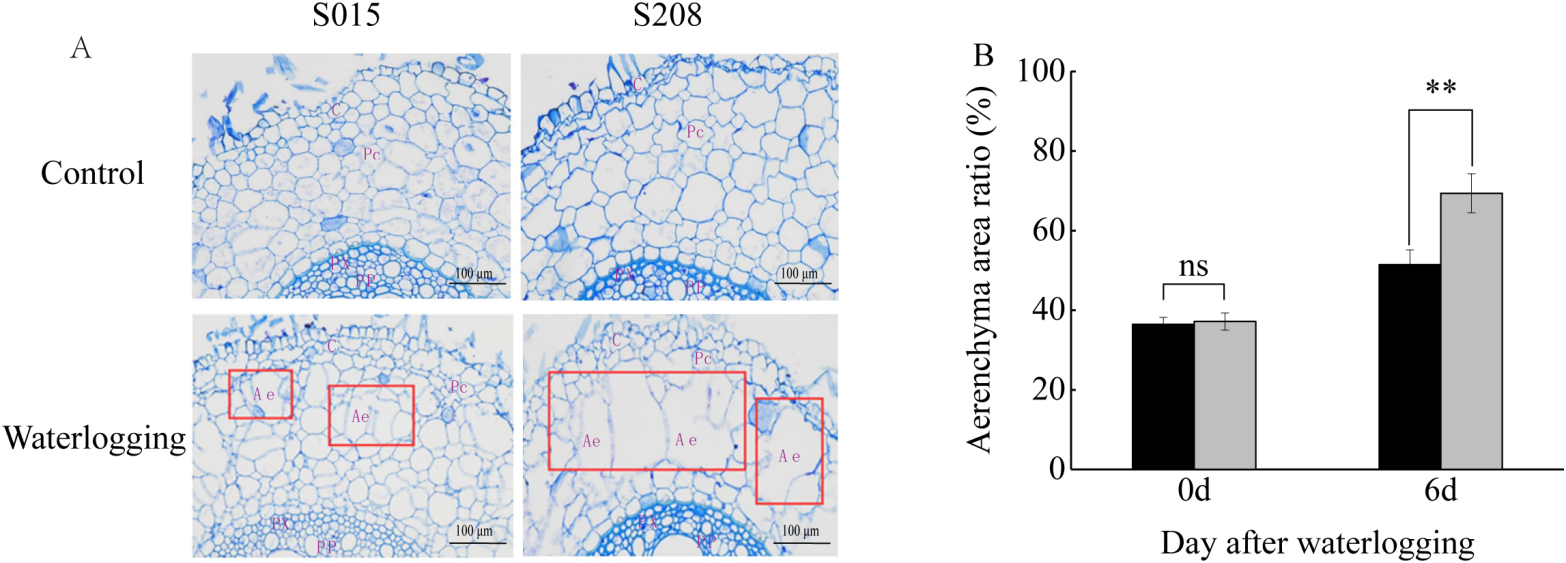
Anatomical features of adventitious roots **(A)** and statistics of aerenchyma in adventitious roots **(B)** in sorghum seedlings after 6 days of waterlogging treatment. C, cortex; Pc, thin-walled cells; Pp, primary phloem; Px, primary xylem; Ae, aerenchyma. Results are the mean ± SD. ns represent there was no significant differences, ** represents the significant differences at p < 0.01.

Waterlogging stress triggers cell death and facilitates aerenchyma formation in roots, an adaptative response to hypoxic conditions. To assess aerenchyma formation in sorghum adventitious roots, transverse sections were obtained 10 mm from the root tips after 6 days of waterlogging. Observation of paraf-fin-embedded root sections revealed that under waterlogging, cortical parenchyma cells exhibited larger lysed zones compared to the control, where intercellular spaces remained limited. Notably, after 6 days of waterlogging, the proportion of aerenchyma tissue was significantly greater in ‘S208’ than in ‘S015’, suggesting a more pronounced structural adaptation to hypoxia in ‘S208’ (**Figure 2**).

### 3.3 Anaerobic respiratory enzyme activity analysis of different sorghum varieties under waterlogging treatment

**Figure 3:**
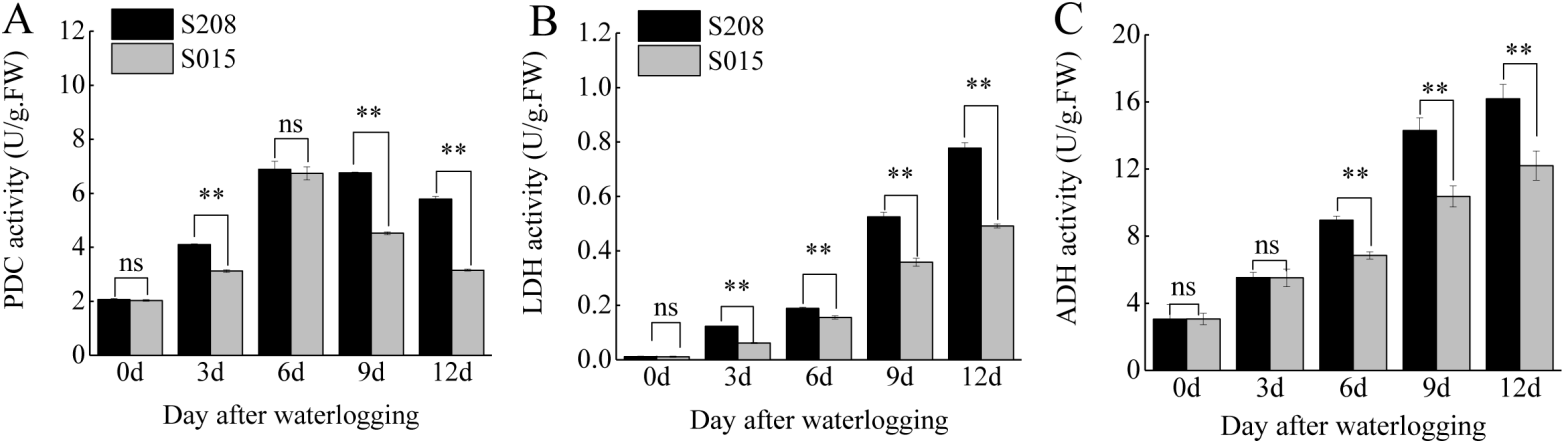
Effect of anaerobic respiratory enzyme activity in root between ‘S208’ and ‘S015’. **(A)** Pyruvate decarboxylase (PDC) activity. **(B)** Lactate dehydrogenase (LDH) activity. **(C)** Alcohol dehydrogenase (ADH) activity. Results are the mean ± SD. ns represent there was no significant differences, ** represents the significant differences at p < 0.01.

Waterlogging stress activates anaerobic respiration in plants, and the activities of key enzymes involved in this pathway were measured in the roots of ‘S208’ and ‘S015’. Significant differences in the activities of pyruvate decarboxylase (PDC), Lactate dehydrogenase (LDH) activity and alcohol dehydrogenase (ADH) were observed between the two genotypes under waterlogging conditions (**Figures 3A–C**).

In both genotypes, the activities of anaerobic respiratory enzymes increased under waterlogging stress, but the increases were more pronounced in ‘S208’. In ‘S208’, PDC activity increased by 1.98-fold, 3.33-fold, 3.27-fold, and 2.80-fold compared to the control at 3, 6, 9, and 12 days of waterlogging, respectively. In contrast, the increases in ‘S015’ were 1.54-fold, 3.31-fold, 2.22-fold, and 1.55-fold at the same time points. LDH activity in ‘S208’ roots increased by 10.48-fold, 16.10-fold, 44.72-fold, and 66.26-fold at 3, 6, 9, and 12 days, respectively, while the corresponding increases in ‘S015’ were 5.53-fold, 13.93-fold, 32.10-fold, and 44.06-fold. Similarly, ADH activity in ‘S208’ in-creased by 1.81-fold, 2.93-fold, 4.68-fold, and 5.30-fold at 3, 6, 9, and 12 days, respectively, compared to increases of 1.80-fold, 2.23-fold, 3.38-fold, and 3.98-fold in ‘S015’. These results indicate that the anaerobic respiratory enzyme activities in ‘S208’ were consistently higher than those in ‘S015’, contributing to its superior tolerance to waterlogging stress.

### 3.4 Analysis of sorghum root transcriptome under waterlogging stress

To investigate the molecular mechanisms underlying sorghum’s response to waterlogging stress, roots from four treatment groups, ‘S015’-CK (control), ‘S015’-W (waterlogging), ‘S208’-CK (control), and ‘S208’-W (waterlogging), were collected after 24 hours of waterlogging stress. This time point captures the early transcriptional response at the onset of waterlogging stress—a critical window for identifying initial stress-signaling genes and early metabolic adjustments. These early changes lay the foundation for subsequent morphological and physiological adaptations, and thus remain a key component of understanding the full waterlogging response cascade in sorghum. Three biological replicates were included for each sample, resulting in a total of 12 libraries. The transcriptomes of the sorghum plants were sequenced using the high-throughput Illumina sequencing platform, and at least 6.51 Gb of clean bases were obtained per sample. Low-quality reads were filtered out based on base quality values, with all samples achieving Q20 scores above 98.03% and Q30 scores above 94.00%. The GC content was greater than 50.58% (**Supplementary Table 1**). This indicates high-quality sequencing data suitable for subsequent analysis.

**Figure 4:**
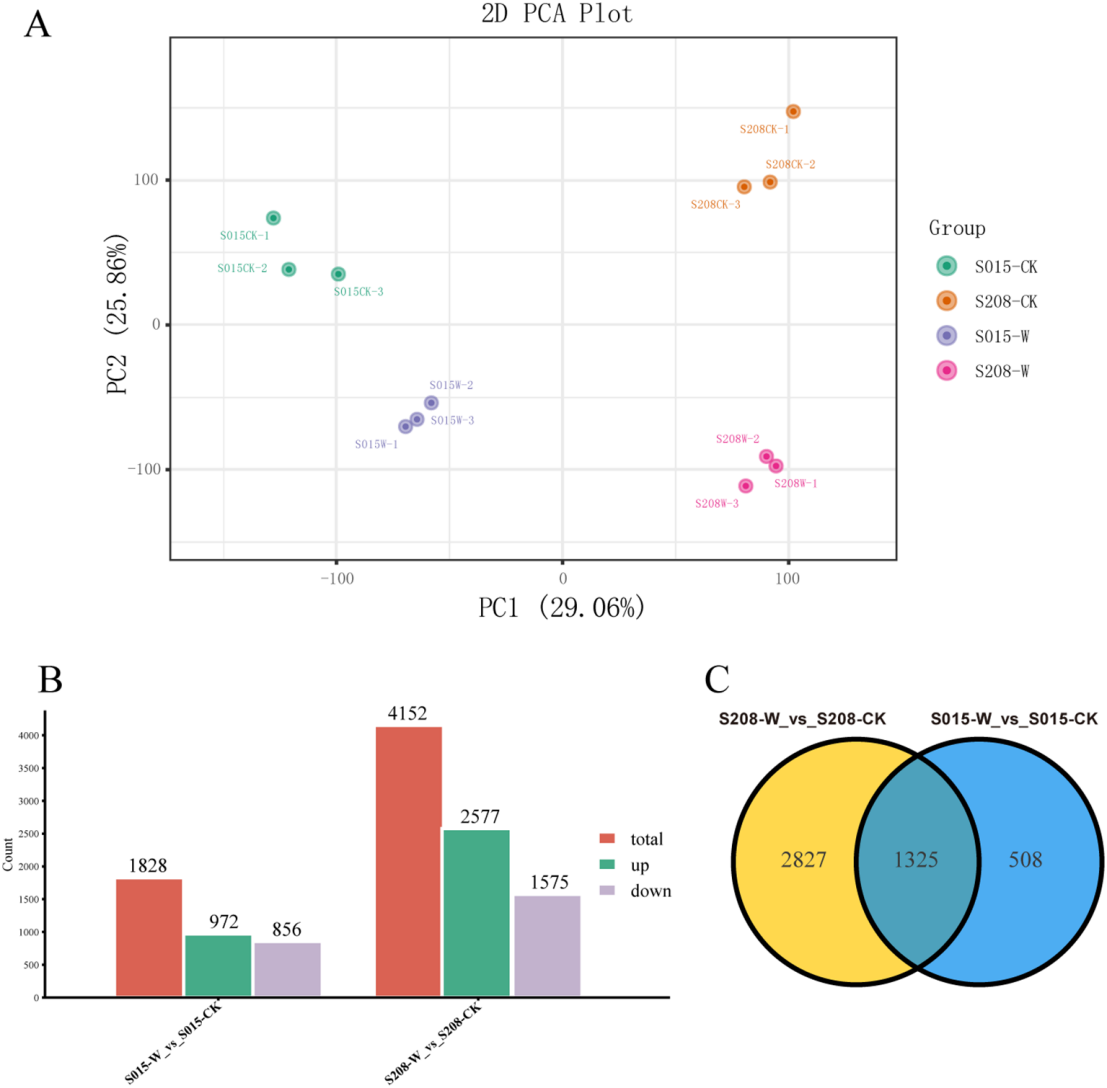
Transcriptome analysis in roots of ‘S208’ and ‘S015’ under control and waterlogging conditions. **(A)** Principal component analysis (PCA) of transcript changes separates the samples under control and waterlogging conditions. **(B)** The DEGs, including up-regulated and down-regulated, in in roots of ‘S208’ and ‘S015’ under control and waterlogging conditions. **(C)** Venn diagram depicting the number of all DEGs expressed in root tissues of ‘S208’ and ‘S015’ genotypes under stress and control conditions.

Principal component analysis (PCA) was performed on the transcriptome data, as shown in **Figure 4A**. The PCA plot revealed that the three replicates for each of the two waterlogging-tolerant genotypes (‘S015’ and ‘S208’) under both control and waterlogging treatments clustered closely together, suggesting good reproducibility and high-quality transcriptome data. This confirmed that the sequencing data was reliable and met the requirements for further bioinformatics analyses.

DEGs between the different treatments were identified using the Fragments Per Kilobase of exon model per Million mapped fragments (FPKM) method. Genes with a log2 fold change ≥ 1 and a p-value ≤ 0.05 were considered differentially expressed. As shown in **Figure 4B**, a total of 1,828 DEGs were identified in ‘S015’-W vs ‘S015’-CK, with 972 genes down-regulated and 856 genes up-regulated. In the comparison be-tween ‘S208’-W and ‘S208’-CK, 4,252 DEGs were identified, of which 1,575 genes were up-regulated and 2,577 genes were down-regulated.

Moreover, 508 DEGs were uniquely found in ‘S015’ under 24 hours of waterlogging stress, while 2,827 DEGs were unique to ‘S208’ under the same conditions. A total of 1,325 DEGs were common between the two genotypes (**Figure 4C**). These results suggest distinct transcriptional responses to waterlogging stress in ‘S015’ and ‘S208’, with specific genes differentially regulated in each genotype.

### 3.5 Gene ontology enrichment analysis of DEGs

To investigate the function of DEGs in sorghum under waterlogging stress, GO enrichment analysis was performed. The differential genes from both sorghum varieties after waterlogging stress were classified into three main categories: cellular component, molecular function, and biological process.

**Figure 5:**
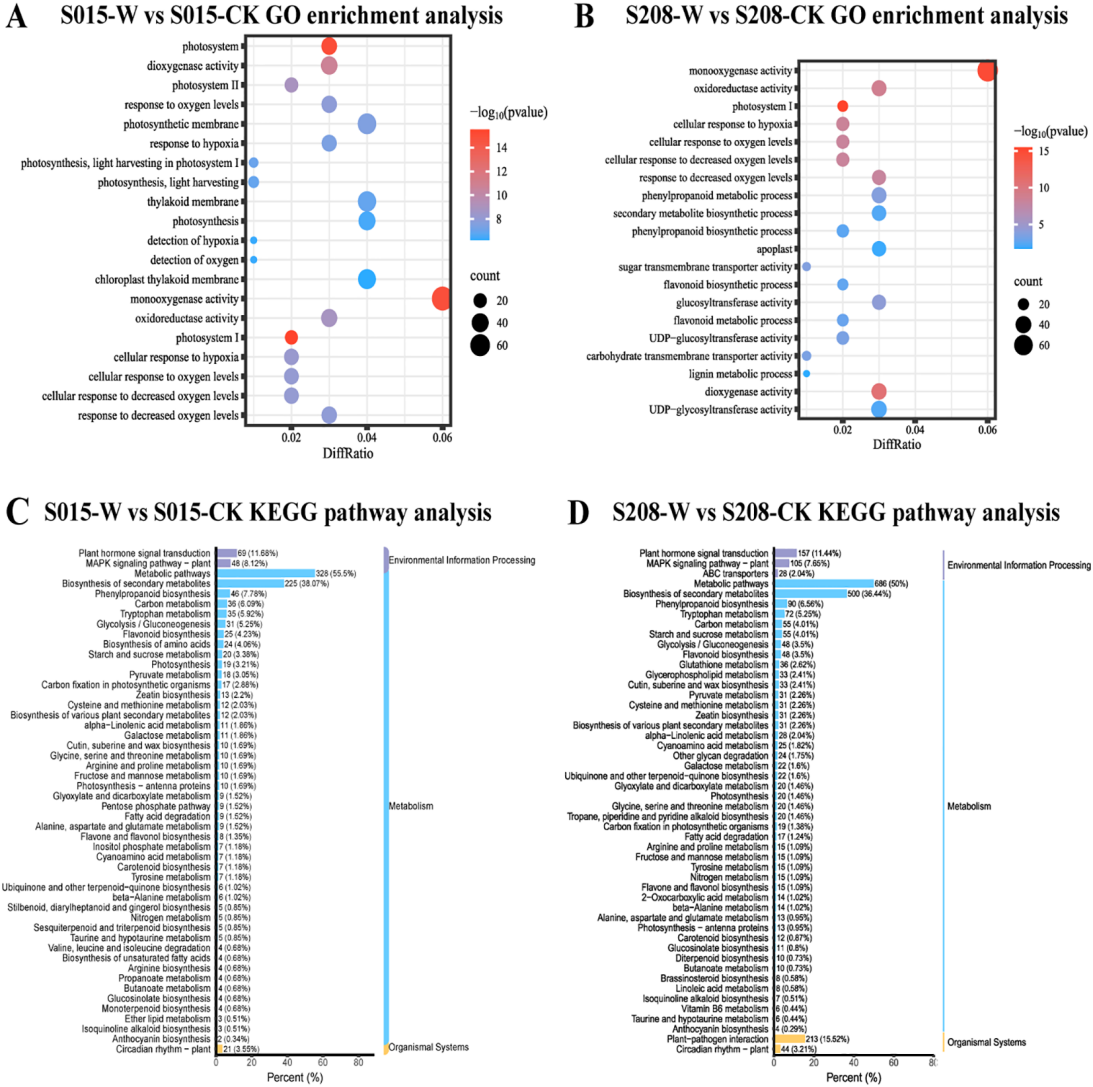
GO enrichment barplot and KEGG enrichment scatterplot of the waterlogging responsive DEGs in ‘S208’ and ‘S015’, **(A)** ‘S015’-W vs ‘S015’-CK GO enrichment analysis. **(B)** ‘S208’-W vs ‘S208’-CK GO enrichment analysis. **(C)** ‘S015’-W vs ‘S015’-CK KEGG pathway analysis. **(D)** ‘S208’-W vs ‘S208’-CK KEGG pathway analysis.

Significant enrichment was observed in genes related to oxygenase activities, including both monooxygenase and dioxygenase activities, in both sorghum varieties after waterlogging stress. Furthermore, GO terms such as “response to hypoxia” (GO:0071456) and “response to oxygen levels” (GO:0036294) were significantly enriched in ‘S015’-W vs ‘S015’-CK. In contrast, molecular functions like UDP-glucosyltransferase activity (GO:0035251), phenylpropanoid metabolism (GO:0009698), and flavonoid metabolism (GO:0009812) were notably enriched in ‘S208’-W vs ‘S208’-CK (**Figures 5A, B**).

KEGG pathway enrichment analysis was performed. The results showed that the differential genes in both sorghum varieties under waterlogging stress and control conditions were mainly enriched in energy metabolism and hormonal pathways. These pathways included plant hormone signal transduction (ko04075), secondary metabolite biosynthesis (ko01110), phenylpropanoid biosynthesis (ko00940), pyruvate and glucose metabolism (ko00620), carbon metabolism (ko01200), glycolysis (ko00010), starch and sucrose metabolism (ko00500), zeatin biosynthesis (ko00908), galactose metabolism (ko00052), and carotenoid biosynthesis (ko00906).

However, certain pathways were more significantly enriched in specific varieties. For example, pathways related to unsaturated fatty acid biosynthesis (ko01040), petose phosphate pathway (ko00030), and amino acid biosynthesis (ko01230) were enriched in ‘S015’-W vs ‘S015’-CK, whereas pathways such as glycerophospholipid metabolism (ko00564), oleoresin lactone biosynthesis (ko00905), and plant-pathogen interaction (ko04626) were enriched in ‘S208’-W vs ‘S208’-CK. These findings suggest that waterlogging-tolerant strains of sorghum exhibit distinct enrichment patterns in KEGG pathways under waterlogging stress.

Next, we focused on the expression of DEGs in significantly enriched pathways, such as starch and sucrose metabolism, biosynthesis of plant secondary metabolites, phenylpropanoid biosynthesis, glycolysis, and plant hormone signal transduction. The analysis revealed that the expression of differential genes in these pathways was generally lower in ‘S015’ than in ‘S208’ under waterlogging stress. For instance, in the biosynthesis of secondary metabolites, ‘S015’ had 225 differential genes under waterlogging stress, whereas ‘S208’ had 500 genes involved in the same process.

In starch and sucrose metabolism, under waterlogging stress, the number of up-regulated genes in ‘S015’ was 6 and that of down-regulated genes was 14, while in ‘S208’, the numbers were 16 and 39, respectively. In the plant hormone signal transduction pathway, ‘S015’ exhibited the up-regulation of 30 genes and the down-regulation of 39 genes, whereas ‘S208’ showed the up-regulation of 55 genes and the down-regulation of 102 genes (**Figures 5C, D**).

### 3.6 Analysis of differential expressed genes related to energy metabolism, hormone regulation and cell wall modification under waterlogging stress

**Figure 6:**
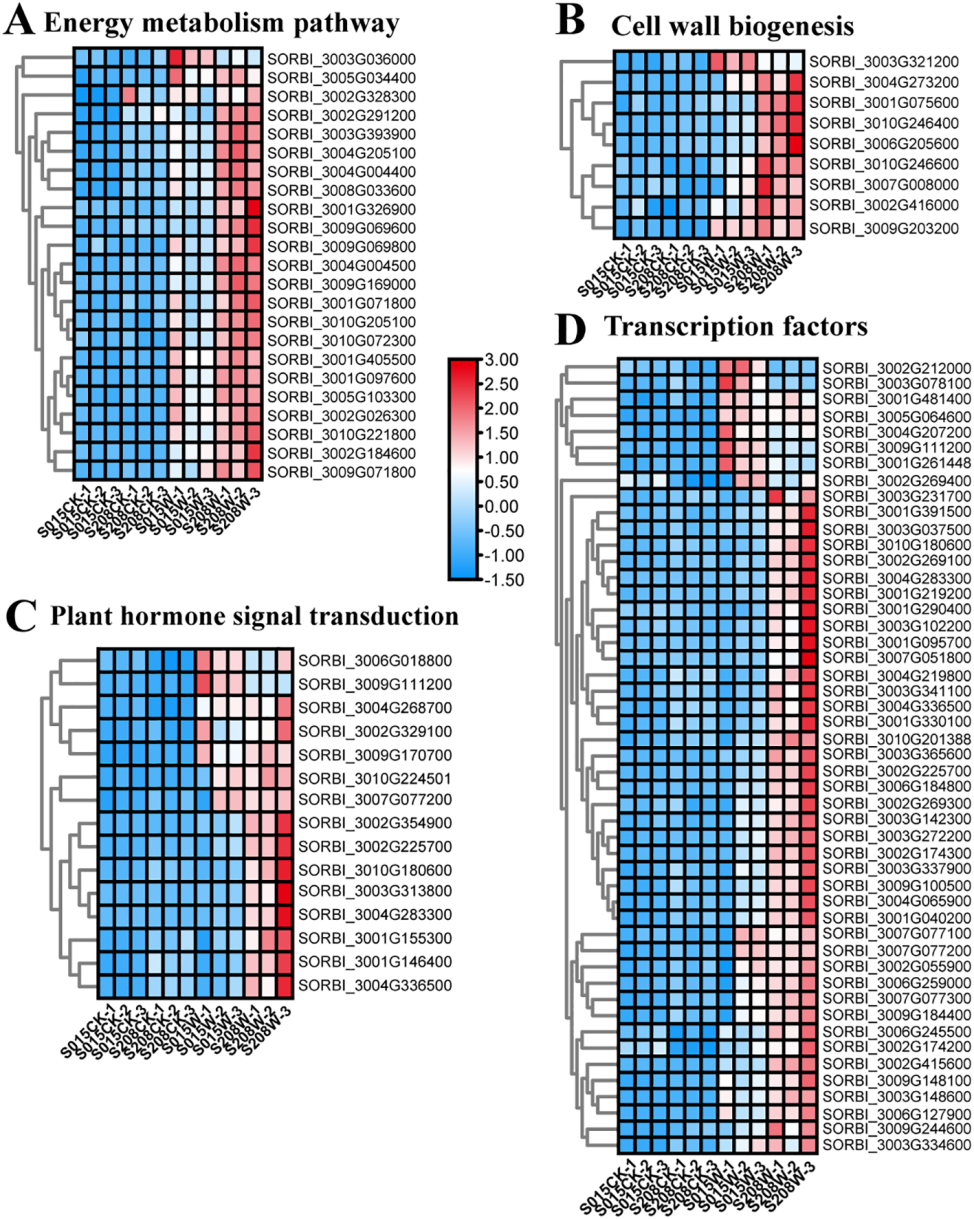
Heatmap of waterlogging responsive DEGs in ‘ ‘S208’ and ‘ ‘S015’’, **(A)** DEGs related energy metabolism pathway. **(B)** DEGs related to cell wall organization or biogenesis. **(C)** DEGs related to plant hormone signal transduction. **(D)** DEGs related to transcription factors. The values of the blue to red gradient bar indicate the change in expression.

Energy deprivation due to hypoxia is one of the major factors affecting the survival of waterlogged plants. In this study, KEGG enrichment analysis showed that metabolic pathways such as starch and sucrose metabolism (SSM), pyruvate metabolism (PYM), and glycolysis (GLY) were significantly enriched in the differential genes of both sorghum varieties. Further analysis of key genes in these metabolic pathways revealed that genes encoding enzymes such as sucrose synthase (SUS) 1 (*SORBI_3010G072300*), trehalose-6-phosphatase (T6P) (*SORBI_3002G184600*), ADH1 isoform X1 (*SORBI_3001G097600*), ADH2 (*SORBI_3005G103300*), pyruvate kinase (PK) (*SORBI_3005G034400*), and PK (*SORBI_3001G326900*) were significantly expressed after waterlogging stress. Expression levels of these genes were higher in ‘S208’ compared to ‘S015’, indicating that ‘S208’ exhibits stronger anaerobic respiratory enzyme activity, which aligns with the anaerobic respiratory enzyme activity measurement results (**Figure 6A**). The upregulation of key genes in 24h ‘S208’ roots, including *SUS1* (*SORBI_3010G072300*, involved in sucrose breakdown) and *ADH2* (*SORBI_3005G103300*, a core anaerobic respiration enzyme), provides a molecular basis for the subsequent physiological advantages observed at 3d-12d. For example, the early induction of *SUS1* in ‘S208’ (24h post-waterlogging) likely enhances carbohydrate availability, supporting the significant increase in adventitious root number (128.57% higher than CK at 3d). This suggests that the 24h transcriptional response primes ‘S208’ for efficient energy utilization under prolonged hypoxia, underscoring the relevance of early-stage transcriptomic data despite the need for extended time-point analysis.Aerenchyma formation enhances aeration between roots and stems, increasing oxygen availability and helping maintain normal root function. This process is a crucial mechanism for plant adaptation to waterlogging stress. We identified several DEGs associated with aerenchyma formation, including *xyloglucan endotransglycosidases* (*XTH22*, *XTH24*, *XTH30*), *xyloglucan glycosyltransferases* (*XGT9*), and *pectin esterases* (*PE, PEI8, PE53 isoform X2*). These genes were significantly up-regulated after waterlogging, with their expression being more pronounced in ‘S208’-W than in ‘S015’-W. For example, the expression of *XTH24* in ‘S208’-W was 3.4 times higher than in ‘S015’-W. Additionally, aerenchyma formation was significantly greater in ‘S208’than in ‘S015’ after waterlogging treatment (**Figure 6B**).

Ethylene is a key hormone involved in the plant response to waterlogging. Among the DEGs related to ethylene signaling, one ethylene receptor gene (*ETR2*) and four ethylene-responsive transcription factors (*ERF4*, *ERF24*, *ERF71*, and *ERF109*) were significantly up-regulated after waterlogging. Besides ethylene, DEGs associated with other hormones, such as cytokinins (CTK), gibberellins (GA), and abscisic acid (ABA), were also identified. Notably, genes such as the *abscisic acid receptor* (*PYL3L*), *abscisic acid 8’-hydroxylase 1* (*SORBI_3004G268700*), and the *key rate-limiting enzyme for ABA bio-synthesis* (*NCED1*) were significantly up-regulated after waterlogging stress. Further-more, gibberellin-responsive protein 2 and several growth hormone-responsive proteins, including *IAA9X2*, *IAA23*, and *SAUR71*, were identified and up-regulated after water-logging, with expression levels being significantly higher in ‘S208’-W than in ‘S015’-W (**Figure 6C**).

Transcription factors (TFs) play a critical role in plant responses to environmental stress. In response to adversity, plants activate TFs via signal transduction pathways to enhance resistance by inducing downstream expression of stress-related genes. In this study, out of 4,655 DEGs, 49 were annotated as transcription factors. The most abundantly annotated TF families were AP2/ERF, AUX, WRKY, bHLH, MYB, NAC, and C2H2. The largest number of DEGs were annotated as ERF transcription factors, with 18 such genes identified. Among these, *ERF109* (*SORBI_3002G225700*), *ERF3-like* (*SORBI_3006G259000*), *ERF071* (*SORBI_3003G148600*), *ERF8* (*SORBI_3009G184400*), and *ERF11* (*SORBI_3007G077100*) were highly up-regulated after waterlogging. Addition-ally, eight WRKY genes were identified, with *WRKY71* (*SORBI_3004G065900*) showing a more pronounced up-regulation in ‘S208’ after waterlogging treatment. In the MYB family, eight genes were identified, including *MYB4* (*SORBI_3001G219200*) and *MYB44* (*SORBI_3010G201388*), both of which were significantly up-regulated after waterlogging and exhibited higher expression levels in ‘S208’ than in ‘S015’. Moreover, three AUX-related genes, four NAC-related genes, and seven bHLH-related genes were also identified (**Figure 6D**).

### 3.7 Gene co-expression analysis by weighted gene co-expression network analysis

**Figure 7:**
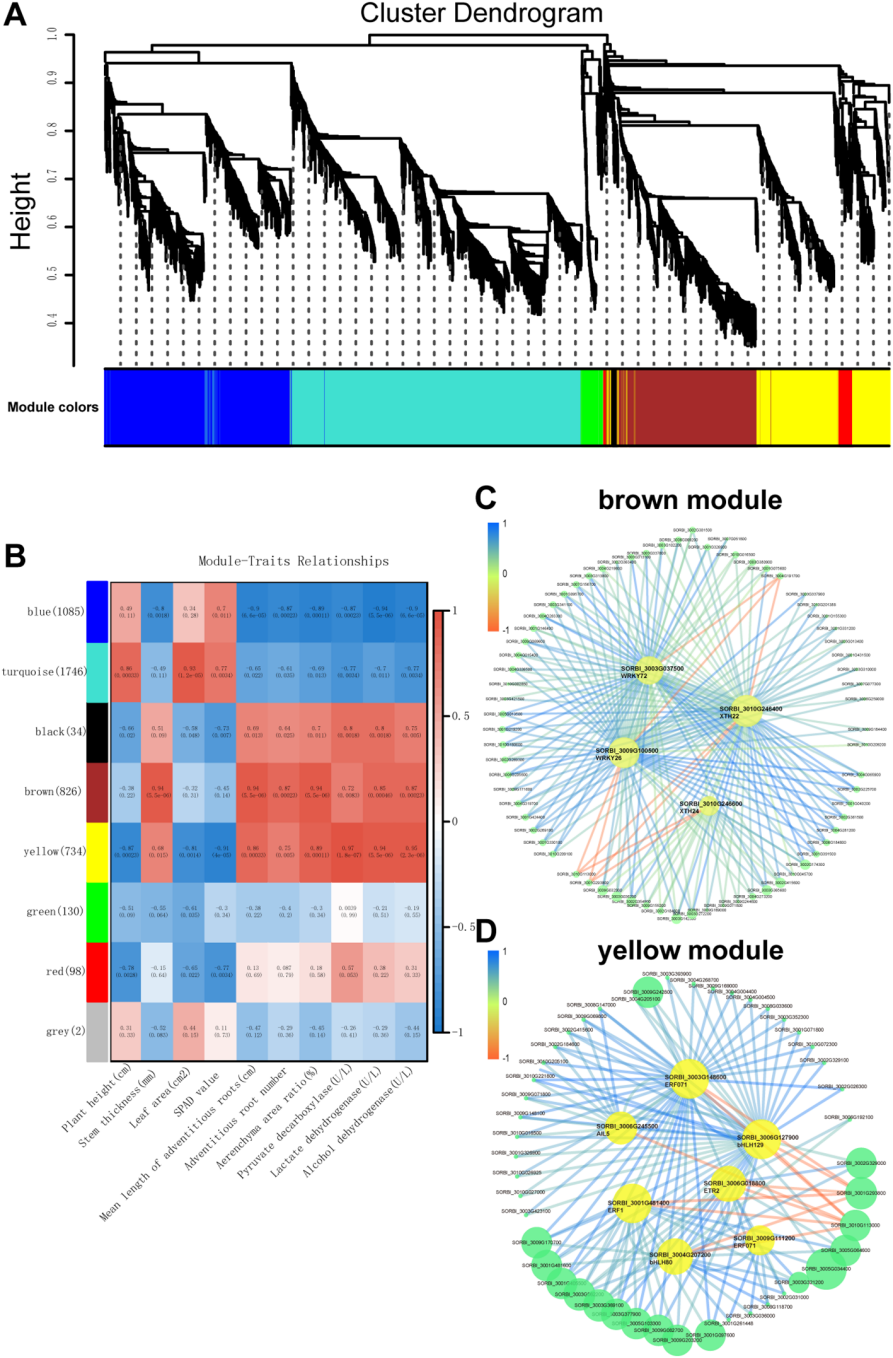
Weighted gene co-expression network analysis (WGCNA) of waterlogging-responsive gene modules in sorghum. **(A)** Clustering dendrogram with dissimilarity based on topological overlap, together with the assigned module colors. **(B)** Correlation between modules and traits. **(C)** Gene interaction network of hub genes in brown module. **(D)** Gene interaction network of hub genes in yellow module.

To identify key regulatory genes involved in the response to waterlogging stress in two sorghum varieties, we performed a weighted gene co-expression network analysis (WGCNA) on 4,655 DEGs. This analysis categorized the genes into 8 distinct modules (**Figures 7A, B**). Among these, the ‘brown’ and ‘yellow’ modules exhibited significant correlations (r > |0.8|) with various physiological and biological traits of sorghum seedlings under waterlogging stress.

The ‘brown’ module showed positive correlations with several key traits, including stem thickness (r = 0.94, p = 5.5e−06), mean adventitious root length (r = 0.94, p = 5.5e−06), adventitious root number (r = 0.87, p = 0.00023), aerenchyma area ratio (r = 0.94, p = 5.5e−06), lactate dehydrogenase activity (r = 0.85, p = 0.00046), and alcohol de-hydrogenase activity (r = 0.87, p = 0.00023) (**Figure 7C**). Similarly, the ‘yellow’ module was positively correlated with mean adventitious root length (r = 0.86, p = 0.00033), aerenchyma area ratio (r = 0.89, p = 0.00011), pyruvate decarboxylase activity (r = 0.97, p = 1.8e−07), lactate dehydrogenase activity (r = 0.94, p = 5.5e−06), and alcohol dehydro-genase activity (r = 0.95, p = 2.3e−06) (**Figure 7D**).

Gene network visualization of the ‘brown’ and ‘yellow’ modules revealed several candidate hub genes. In the ‘yellow’ module, the hub genes included *SORBI_3003G148600* (*ERF071*), *SORBI_3006G245500* (*AIL5*), *SORBI_3006G018800* (*ETR2*), *SORBI_3001G481400* (*ERF1*), *SORBI_3009G111200* (*ERF071*), *SORBI_3006G127900* (*bHLH129*), and *SORBI_3004G207200* (*bHLH80*). In the ‘brown’ module, the hub genes identified were *SORBI_3003G037500* (*WRKY72*), *SORBI_3009G100500* (*WRKY26*), *SORBI_3010G246400* (*XTH22*), and *SORBI_3010G246600* (*XTH24*). These hub genes likely play critical roles in regulating the expression of other genes within their respective modules, thereby contributing to the plant’s response to waterlogging stress.

### 3.8 Quantitative real-time fluorescence validation

**Figure 8:**
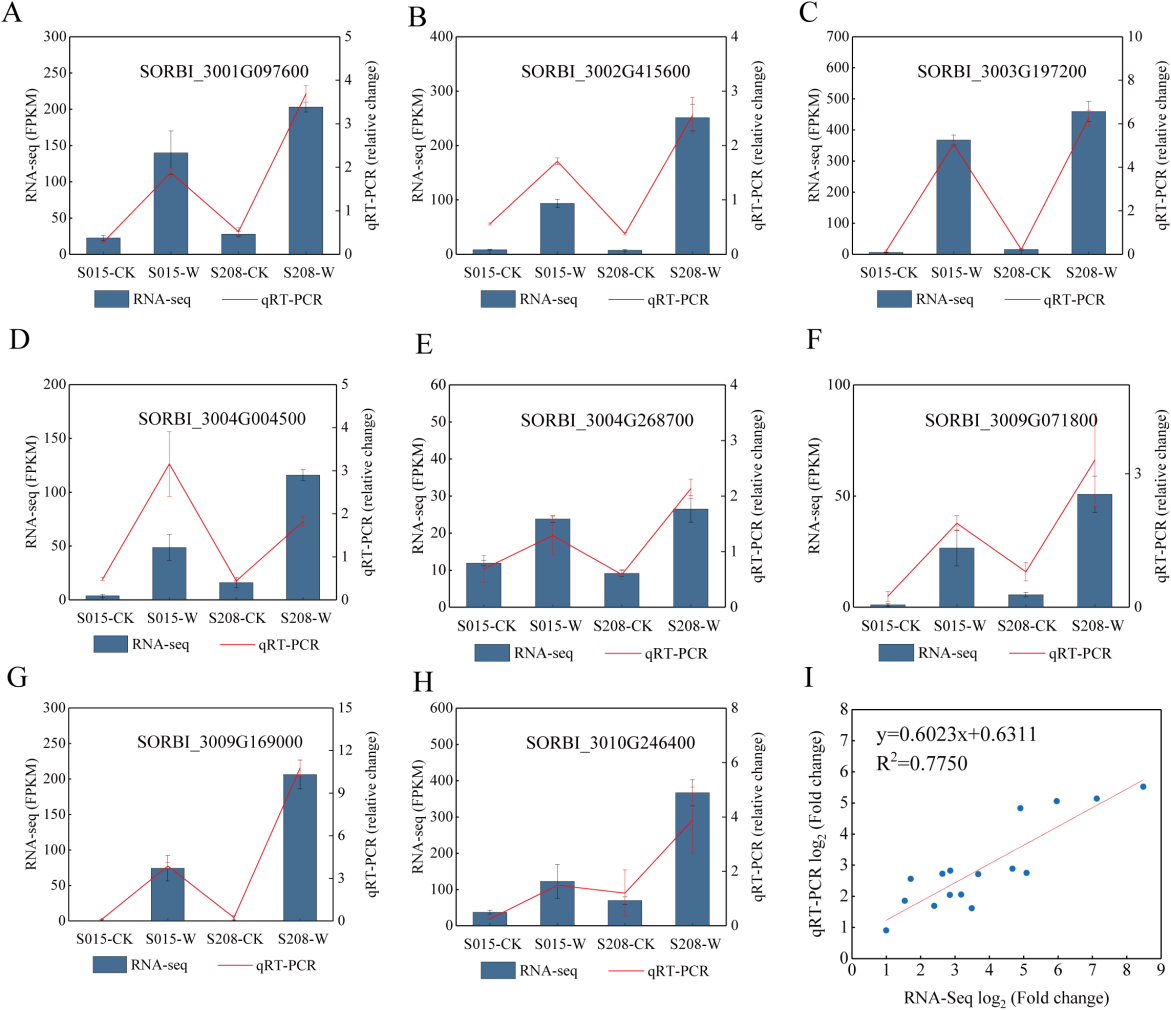
Validation of DEGs in the RNA-seq results via qRT-PCR. **(A–H)** The expression levels of 8 genes determined by RNA-seq and qRT-PCR are presented on the left and right Y-axis, re-spectively. The columns and red lines represent the results of the RNA-seq and qRT-PCR analyses, respectively, and the error bars represent the means ± SE values (n = 3). **(I)** The correlation be-tween the log2 (fold change) of the 8 DEGs from the RNA-seq (X-axis) and qRT-PCR (Y-axis) analyses.

To validate the accuracy of the transcriptome sequencing, eight genes related to waterlogging stress were randomly selected for qRT-PCR analysis. A significant positive correlation was observed between the qRT-PCR and RNA sequencing data (R² = 0.78) (**Figure 8**). This result demonstrates the accuracy and reliability of the transcriptome sequencing findings.

## 4 Discussion

Waterlogging stress imposes severe hypoxia on root systems, distrupting aerobic respiration, and leading to energy depletion, metabolic imbalance, and impaired growth (Tong et al., 2021; Xu et al., 2023). Sorghum, as a crop with high genetic diversity, offers valuable germplasm resources that can be utilized to improve stress tolerance through the identification and application of favorable genes. In this study, we integrated morphological, phsiological, and transcriptomic analyses to elucidate mechanisms of two sorghum genotypes, ‘S015’ (sensitive) and ‘S208’ (tolerant). The results collectively demonstrate that ‘S208’ has stronger waterlogging tolerance and represents a promising genetic resource for breeding waterlogging-resistant sorghum varieties.

### 4.1 Morphological and anatomical adaptations

Waterlogging initial damages the root system, thereby impairing overall plant growth (Xu et al., 2023). Roots are essential for water and nutrients uptake, and the development of adventitious roots and aerenchyma constitutes a key adaptive strategy under waterlogging stress (Zhang et al., 2015; Manik et al., 2022). These morphological and anatomical adaptations help improve oxygen acquisition in submerged tissues, mitigate hypoxic stress, and promote plant survival in waterlogged environments (Xu et al., 2023). In this study, ‘S208’ exhibited faster and more extensive adventitious root formation and a higher proportion of aerenchyma compared with ‘S015’. In maize the adventitious root formation often involves the elongation of pre-existing root primordia (Kaur et al., 2021), whereas in ‘S208’, new adventitious roots emerged more rapidly from the stem base. This difference may reflect genetic and growth habit variations between the two varieties.

### 4.2 Comparison of physiological responses

Waterlogging-induced hypoxia triggers a range of physiological and metabolic changes, including decreased metabolite levels, growth inhibition, and acitivation of anaerobic respiration pathways (Barickman et al., 2019; Zhang T. et al., 2023). Under waterlogging, ATP required for root activity is produced mainly through fermentation and glycolysis rather than oxidative phosphorylation (Omoarelojie et al., 2021). Previous studies have shown that that loss-of-function mutants of *ADH*, *PDC* and *LDH* in Arabidopsis exhibit reduced survival under low oxygen conditions (Ventura et al., 2020). In the present study, the activities of PDC, LDH and ADH increased significantly under waterlogging stress, consistent with reports in barley (Luan et al., 2023), cucumber (Chen et al.), and Kiwifruit (Liu et al., 2022). Notably, all three enzyme activities were higher in ‘S208’ than in ‘S015’, suggesting that enhanced anaerobic respiratory activity may contribute to the superior waterlogging tolerance of ‘S208’. Elevated activities of these enzymes likely help maintain NAD+ regeneration and ATP production under hypoxic, supporting adventitious root elongation and sustaining root function and plant growth.

### 4.3 Comparison of molecular mechanisms

To further explore the molecular basis of waterlogging tolerant in sorghum, we performed transcriptome sequencing of roots under waterlogging stress. GO and KEGG enrichment analyses highlighted differential expressed genes associated with energy metabolism, hormone regulation and cell wall modification.

Energy deficiency caused by hypoxia is a major constraint on plant survival under waterlogging. Starch serves as a primary energy reserve in plants, and starch and sugar metabolism are crucial for responses to waterlogging stresses. Sucrose synthase (SUS) plays a key role in sucrose breakdown, sustaining sugar supply during hypoxia (Kumutha et al., 2008). Previous studies have reported up-regulation of *SUS* genes under low-oxygen conditions (Baud et al., 2004; Wang et al., 2021), and overexpression of *SUS* in cucumber enhances hypoxia tolerance (Wang et al., 2014). Here, the expression of *sucrose synthase 1* (*SUS*, *SORBI_3010G072300*) was significantly higher in ‘S208’ than in ‘S015’ under waterlogging. Moreover, *treha-lose-6-phosphatase* (*T6P, SORBI_3002G184600*), which helps maintain metabolic homeostasis in response to sucrose availability (Schluepmann et al., 2003), was also more highly expressed in ‘S208’. Increased T6P activity has been linked to improved anaerobic germination tolerance in rice (Kretzschmar et al., 2015). These findings suggest that ‘S208’ enhances carbohydrates breakdown to accumulate more energy, thereby improving adaptation to waterlogging. Glycolysis and anaerobic respiration serve as major energy sources under hypoxic. Transctriptome analysis revealed upregulation of *pyruvate decarboxylase* (*PDC*) and *alcohol dehydrogenase* (*ADH*) genes in both genotypes, with higher induction in ‘S208’. Overexpression of *ADH* or *PDC* has been shown to improve waterlogging tolerance in various species (Luo et al., 2017; Luan et al., 2023). The stronger induction of *ADH* (*SORBI_3009G069600*) in ‘S208’ further underscores the importance of anaerobic respiration in waterlogging adaptation.

Aerenchyma formation enhances oxygen diffusion to roots and is linked to cell wall modifications. Several DEGs encoding cell wall-modifying enzymes-such as *xyloglucan endotransglycosidases* (*XTH22*, *XTH24*, *XTH30*) and *xyloglucan glycosyltrans-ferases* (*XGT9*) were up-regulated under waterlogging. Notably, *XTH24* expression was 3.4-fold higher in ‘S208’ than in ‘S105’, suggesting more active aerenchyma formation in ‘S208’.

Phytohormones, especially ethylene, are central regulators of waterlogging responses. We identified DEGs related to ethylene, auxin, cytokinin, and ABA signaling. Upregulation of *ABA receptor gene* (*PYL3L*) and *ABA biosynthesis genes* (*NCED1*) implies ABA involvement in waterlogging adaptation (Zhang et al., 2022). Higher expression of IAA-responsive genes in ‘S208’ also suggests that auxin signaling may promote adventitious root development, further enhancing tolerance.

### 4.4 Transcriptional regulation and network integration

Transcription factors (TFs) are important in plant stress adaptation. For instance, ERF-VII members *AcERF74* and *AcERF75* up-regulate *AcADH1* under waterlogging in kiwifruit (Liu et al., 2022; Liu et al., 2023), and *ZmEREB180* overexpression improved waterlogging tolerance by promoting adventitious rooting and ROS homeostasis (Yu et al.). In Arabidopsis, a RAP2.2 and WRKY module aids hypoxia adaptation (Tang et al., 2021), with *WRKY33* or *WRKY12* overexpressing increasing hypoxia resistance. Wang et al (Wang et al., 2023). reported that WRKY, MYB, bHLH, NAC, ERF, DOF, HD-ZIP and DBP TFs participated in waterlogging response. Our transcriptome data indicated that AP2/ERF, WRKY, bHLH, MYB, and NAC families are involved in sorghum’s response to waterlogging. Weighted gene co-expression net-work analysis (WGCNA) identified hub genes such as *ERF071* and *WRKY72* as key regulators. It should be noted that our experimental design presents limitations for WGCNA, which typically requires larger sample size and more traits to ensure module robustness. To reduce bias, we implemented strict RNA-seq data quality, focused on modules strongly correlated with physiological traits (|r| > 0.8; e.g., aerenchyma ratio and ADH activity), and validated hub genes (e.g., ERF071, WRKY72) via qRT-PCR (**Figure 8**), with their expression patterns consistent with WGCNA results. Compared to well-studied species like rice, where *SUBMERGENCE1A* regulates anaerobic metabolism and adventitious rooting (Alpuerto et al., 2016), sorghum appears to employ distinct TF regulatory patterns. Although ERF and WRKY are widely implicated in waterlogging response, the specific genes and their expression patterns may differ. For example, *ERF071* in sorghum may coordinate multiple response pathways related to energy metabolism and aerenchyma formation, differing from the role of *SUB1A* in rice. These TFs likely regulate adventitious root development, aerenchyma formation, and anaerobic respiration, providing new insights into the molecular basis of waterlogging tolerance in sorghum.

Overall, this study provides a mechanistic framework linking early molecular events to late-stage physiological and morphological outcomes. The tolerant genotype ‘S208’ achieves waterlogging resistance through coordinated activation of energy metabolism, hormone regulation, and cell wall remodeling pathways, governed by central transcriptional regulators such as ERF071 and WRKY72. These findings not only advance our understanding of sorghum’s adaptive responses to hypoxia but also offer valuable genetic targets for breeding waterlogging-tolerant cultivars.

## 5 Conclusions

Waterlogging stress disrupts root aeration and energy metabolism, posing a major constraint on plant growth. In this study, the sorghum genotype ‘S208’ exhibited markedly stronger tolerance than ‘S015’, as evidenced by enhanced growth performance, greater adventitious root formation, and higher aerenchyma development under hypoxic conditions. These morphological traits were supported by elevated activities of anaerobic enzymes (PDC, LDH, ADH) and the early transcriptional activation of energy metabolism genes such as SUS1 and ADH2. Transcriptomic analysis further revealed that S208 rapidly activates hormone- and cell wall–related genes (ERF071, WRKY72, XTH24, XGT9), linking molecular regulation to structural and physiological adaptation. WGCNA identified ERF071 and WRKY72 as key hub regulators that may coordinate energy metabolism, hormone signaling, and anatomical remodeling. Together, these findings outline a hierarchical adaptive mechanism in ‘S208’ involving early transcriptional priming, sustained metabolic adjustment, and morphological plasticity, which collectively confer superior waterlogging tolerance. Although the present transcriptomic analysis was limited to a single time point, future studies integrating multi-time-point and multi-genotype analyses will enable a more comprehensive understanding of the dynamic regulatory networks underlying sorghum waterlogging resistance.

## Data Availability

The data presented in the study are deposited in the Genome Sequence Archive (Genomics, Proteomics & Bioinformatics 2025) in National Genomics Data Center (Nucleic Acids Res 2025), China National Center for Bioinformation / Beijing Institute of Genomics, Chinese Academy of Sciences (GSA: CRA033959), publicly accessible at https://ngdc.cncb.ac.cn/gsa.
