# Supplementary material for: Integrated morphological, physiological, and transcriptomic analyses uncover the mechanisms of waterlogging tolerance in Sorghum bicolor (L.)
Source: Front Plant Sci. 2025 Dec 8;16:1706603. doi: 10.3389/fpls.2025.1706603 (PMC12719275; doi:10.3389/fpls.2025.1706603)
Supplement: Supplementary file 1 [file Table1.docx]

| Sample | Raw Reads | Clean Reads | Clean Base(G) | Error Rate(%) | Q20(%) | Q30(%) | GC (%) |
| --- | --- | --- | --- | --- | --- | --- | --- |
| S015CK-1 | 49944096 | 47738606 | 7.16 | 0.02 | 98.15 | 94.53 | 52.08 |
| S015CK-2 | 50531982 | 48051130 | 7.21 | 0.02 | 98.21 | 94.66 | 52.06 |
| S015CK-3 | 46100296 | 43713540 | 6.56 | 0.03 | 98.03 | 94.23 | 51.95 |
| S208CK-1 | 55597682 | 52934026 | 7.94 | 0.02 | 98.21 | 94.66 | 53.41 |
| S208CK-2 | 56821134 | 54020020 | 8.10 | 0.02 | 98.10 | 94.43 | 53.28 |
| S208CK-3 | 54725028 | 52439666 | 7.87 | 0.03 | 97.97 | 94.00 | 52.93 |
| S015W-1 | 48481464 | 46053910 | 6.91 | 0.02 | 98.23 | 94.74 | 51.88 |
| S015W-2 | 49121668 | 46194660 | 6.93 | 0.02 | 98.28 | 94.85 | 52.27 |
| S015W-3 | 47720088 | 45349820 | 6.80 | 0.02 | 98.22 | 94.65 | 51.85 |
| S208W-1 | 42812740 | 40515688 | 6.08 | 0.02 | 98.12 | 94.46 | 52.50 |
| S208W-2 | 51688264 | 49225902 | 7.38 | 0.02 | 98.18 | 94.60 | 52.63 |
| S208W-3 | 45833846 | 43419990 | 6.51 | 0.02 | 98.20 | 94.66 | 52.26 |

Supplementary Table 1: Transcriptome analysis of 12 sorghum seedling roots.
