# Supplementary material for: Integrated morphological, physiological, and transcriptomic analyses uncover the mechanisms of waterlogging tolerance in Sorghum bicolor (L.)
Source: Front Plant Sci. 2025 Dec 8;16:1706603. doi: 10.3389/fpls.2025.1706603 (PMC12719275; doi:10.3389/fpls.2025.1706603)
Supplement: Supplementary file 2 [file Table2.docx]

Supplementary Table 2: List of primers used for the qRT-PCR analysis

| Gene ID | Forward Primers(5′-3′) | Reverse Primers(5′-3′) | Length |
| --- | --- | --- | --- |
| Sobic.3001G097600 | TAGAGGATTTGGGGAGGGGG | GAAGATCCGAGGGAACACCG | 223 |
| Sobic. 3009G071800 | CGCACCTCACCGACTACCTT | CTCGTCGGGCTGGAAGTAGA | 203 |
| Sobic. 3010G246400 | GCGGCAACTTCTACAAGGACAC | AGAGGTACTCGGACTTGGACTGG | 135 |
| Sobic. 3004G268700 | TACGTCCTTTCACCGCAACA | GGACGAGTAGAGCTGGAACG | 266 |
| Sobic. 3004G004500 | GGGCTCAAGGGATACACGTC | CTAGACCTAGAGGCCGAGGA | 285 |
| Sobic. 3009G169000 | GTTCGGGTGCATCCTCATGT | GAAGATCCGGCGGTAGTTGT | 94 |
| Sobic. 3002G415600 | ACGACGTTCTTCCAGCACCA | GAAAAGCAGCAGCAACAACATG | 259 |
| Sobic. 3003G197200 | TGACGGGTCCCATGGAGATT | TCGTAGTGGCCGTAGACGAA | 187 |
| GAPDH | CCTGTACCGTCCCTCGACTT | ATGCTTGCACCCTGTACTGC | 227 |
